# Activity of temocillin and 15 other agents, including fosfomycin and colistin, against *Enterobacteriaceae* in Hong Kong

**DOI:** 10.1007/s10096-017-3091-8

**Published:** 2017-08-25

**Authors:** Margaret Ip, Christopher K. Lai, Kitty S. C. Fung, K-Tak Wong, Chendi Zhu, Sebastien Van de Velde, Dominic N. Tsang, Peter Hawkey

**Affiliations:** 1 Department of Microbiology, Chinese University of Hong Kong, Prince of Wales Hospital, Shatin, Hong Kong; 2 Department of Pathology, Queen Elizabeth Hospital, Kowloon, Hong Kong; 3 Department of Pathology, United Christian Hospital, Kwun Tong, Hong Kong; 4 Eumedica S.A., Brussels, Belgium; 5 Institute of Microbiology and Infection, University of Birmingham, Birmingham, B15 2TT UK

## Abstract

Limited data are available on temocillin susceptibilities in *Enterobacteriaceae* from Asian countries where antimicrobial resistance is prevalent. The in vitro activities of temocillin and 15 commonly used antimicrobials against 613 non-duplicate blood (*n* = 310) and urine (with clinically significant bacteriuria; *n* = 303) isolates of *Enterobacteriaceae* from patients who attended 3 out of 7 clusters of public hospitals of the Hospital Authority, Hong Kong, during 2015/2016 were tested. Minimum inhibitory concentrations (MICs) were determined by Clinical and Laboratory Standards Institute (CLSI) microbroth dilution (agar dilution with fosfomycin). For temocillin, MICs were also obtained using the British Society of Antimicrobial Chemotherapy (BSAC) microbroth dilution method and interpreted using the BSAC breakpoints. Overall, 93.0% (570) isolates were susceptible to temocillin using BSAC systemic breakpoint (≤8 mg/L) and all except 2 isolates were susceptible using the urinary breakpoint (≤32 mg/L). The extended spectrum beta-lactamase (ESBL) positivity rate was 23.2% (118 out of 508 *E. coli*, *Klebsiella* spp., *Proteus* spp.). Temocillin resistance rate to ESBL-positive isolates was 16.1% using the systemic breakpoint of ≤8 mg/L (MIC_50_ and MIC_90_ were 8 mg/L and 16 mg/L respectively). Two isolates (1 *E. coli*, temocillin MIC 64 mg/L*,* 1 *Klebsiella* sp., MIC 32 mg/mL) were resistant to meropenem and possessed the NDM-5 and KPC-2 genes respectively. Other susceptibility rates were: amoxicillin/clavulanate (59.1%), trimethoprim/sulfamethoxazole (62.5%), ciprofloxacin (71.5%), ceftriaxone (75.4%), nitrofurantoin (76.4%), gentamicin (78.3%), cefepime (81.1%), ceftazidime (83.5%), piperacillin/tazobactam (86%), colistin (88.8%), tigecycline (89.4%), fosfomycin (92.8%), ertapenem (99.0%), amikacin (99.2%) and meropenem (99.7%). Temocillin may be a useful alternative for the treatment of infections caused by ESBL and multi-drug-resistant *Enterobacteriaceae* in Hong Kong, particularly as resistance rates to ciprofloxacin, nitrofurantoin and piperacillin/tazobactam are high.

**Electronic supplementary material:**

The online version of this article (10.1007/s10096-017-3091-8) contains supplementary material, which is available to authorized users

## Introduction

The global pandemic of CTX-M extended spectrum beta-lactamases (ESBLs) has driven rates of multi-drug-resistant Gram-negative bacteria (MDRGNB) to unprecedented levels, particularly in Asia [1]. This is exemplified by recent data reporting an ESBL rate in *E. coli* of 66% in China [2], whereas in Hong Kong, 20% of *E. coli* and 15.4% of *K. pneumoniae* causing bacteraemia in hospitalized patients had been reported to be ESBL-positive isolates [3, 4]. The spread of carbapenemase-producing *Enterobacteriaceae* brought further concerns around carbapenem overuse together with the need for heightened infection control.

Temocillin has been introduced into the UK and a number of European countries for the treatment of infections caused by ESBL-producing *Enterobacteriaceae* [5, 6]. Temocillin, a narrow spectrum penicillin (6-α-methoxy-ticarcillin) with intrinsic stability to AmpCs, ESBLs and some carbapenemases, has been considered potentially a “carbapenem-sparing agent”, especially in the treatment of ESBL-producing enterobacterial infections of the urinary tract [7]. However, limited data exist on the activity of temocillin against *Enterobacteriaceae* in Asian countries and China. We thus sought to evaluate the in vitro activity of temocillin and commonly used antimicrobials (including fosfomycin, tigecycline and colistin) against clinical isolates of *Enterobacteriaceae* in patients with urinary tract infections and/or bacteraemia in Hong Kong hospitals. Most laboratories in this region perform antimicrobial susceptibilities based on the Clinical and Laboratory Standards Institute (CLSI) method, and as the susceptibility to temocillin had been based on the British Society of Antimicrobial Chemotherapy (BSAC)-defined MIC breakpoints for *Enterobacteriaceae* [8], we also examined and compared its activity using the microbroth dilution methods according to the CLSI and the BSAC methods.

## Materials and methods

### Bacterial isolates

Non-duplicate isolates of *Enterobacteriaceae* from blood (*n* = 310) or urine (with clinically significant bacteriuria; *n* = 303) from patients who attended 3 out of 7 clusters of hospitals of the Hospital Authority in Hong Kong between January 2015 and January 2016 were examined. These clusters provide public hospital care with a catchment area for over 50% of the Hong Kong population, and isolates included in this study were from the Prince of Wales Hospital (PWH), North District Hospital (NDH), Shatin Hospital (SH), Queen Elizabeth Hospital (QEH) and United Christian Hospital (UCH). Bacterial identification was based on conventional biochemical methods and/or use of commercial systems, e.g. API, Vitek or MALDI-TOF, as established in each hospital’s accredited microbiology laboratory. The isolates included were *E. coli* (349), *Klebsiella* spp. (109), *Proteus* spp. (50), *Enterobacter* spp. (35), *Citrobacter* spp. (25), *Salmonella* spp. (17), *Morganella* spp. (16), *Serratia* spp. (11), *Providencia* spp. (1).

### Antimicrobial susceptibility testing

The minimum inhibitory concentrations (MICs) to 16 antibiotics, namely, temocillin, amoxicillin/clavulanate, trimethoprim/sulfamethoxazole, ciprofloxacin, nitrofurantoin, gentamicin, amikacin, ceftriaxone, ceftazidime, cefepime, piperacillin/tazobactam, colistin, tigecycline, fosfomycin, ertapenem and meropenem, were performed and interpreted according to the CLSI microbroth dilution method [9, 10]. For fosfomycin, Mueller–Hinton agar supplemented with glucose-6-phosphate was used for MIC determination according to the CLSI agar dilution method [9, 10]. As there are currently no CLSI breakpoints for temocillin, the MICs to temocillin were performed using the BSAC’s microbroth dilution method [11] and interpreted according to the BSAC systemic and urinary breakpoints (≤8 mg/L and ≤32 mg/L respectively) [8]. ESBL detection was confirmed by the combination disks diffusion method according to the CLSI, using cefotaxime/ceftazidime with and without clavulanate [10]. *E. coli* NCTC 10418 and *P. aeruginosa* NCTC 10662 strains were included as controls in the BSAC method [11]. MIC values of temocillin by the BSAC vs CLSI methods were compared and MIC agreement was defined as previously described [12].

## Results

The antimicrobial susceptibilities of 613 isolates of *Enterobacteriaceae* are listed in Supplementary Tables 1 and 2. The susceptibility rates to temocillin according to the BSAC method were 93.0% (570 out of 613) and 100% using the systemic (≤8 mg/L) and urinary (≤32 mg/L) breakpoints respectively. The ESBL positivity rate was 23.2% (118 out of 508 *E. coli*, *Klebsiella* spp., *Proteus* spp.). The temocillin MIC_50_ and MIC_90_ for these ESBL-positive isolates were 8 mg/L and 16 mg/L respectively. The temocillin resistance rate for ESBL-positive isolates was 16.1% using the systemic breakpoint of ≤8 mg/L. Other antimicrobial susceptibility rates were: amoxicillin/clavulanate (59.1%), trimethoprim/sulfamethoxazole (62.5%), ciprofloxacin (71.5%), ceftriaxone (75.4%), nitrofurantoin (76.4%), gentamicin (78.3%), cefepime (81.1%), ceftazidime (83.5%), piperacillin/tazobactam (86%), colistin (88.8%), tigecycline (89.4%), fosfomycin (92.8%), ertapenem (99.0%), amikacin (99.2%) and meropenem (99.7%). Only two isolates (1 *E. coli* (TMO MIC 64 mg/L), 1 *Klebsiella* species (temocillin MIC 32 mg/mL) were resistant to meropenem and possessed the NDM-5 and KPC-2 genes respectively.

**Fig. 1:**
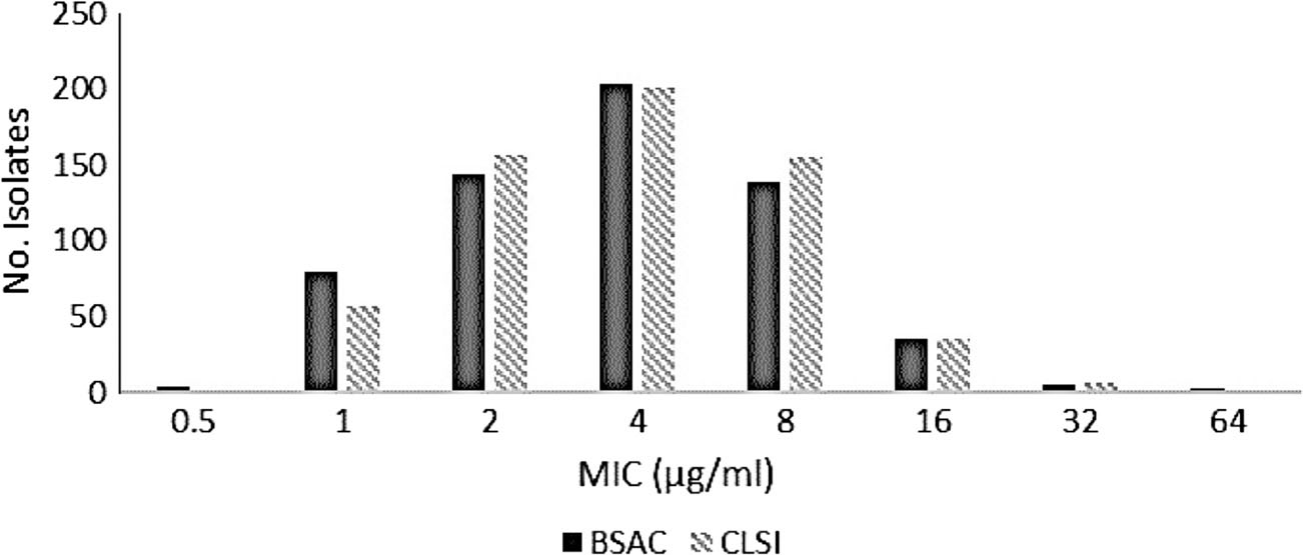
Distribution of temocillin minimum inhibitory concentration values of 613 bacterial isolates according to the British Society of Antimicrobial Chemotherapy (BSAC) and Clinical and Laboratory Standards Institute (CLSI) methods

When MIC values of temocillin obtained by the CLSI and BSAC methods were compared, all MIC values obtained by the CLSI method fell within a two-fold dilution of the MIC values obtained using the BSAC method (Fig. 1). A good correlation, R^2^ = 0.87, was obtained between the two methods and the overall percentage susceptibilities based on the CLSI microbroth method remained unchanged.

## Discussion

Temocillin provides a therapeutic option in the management of infections caused by multi-drug-resistant *Enterobacteriaceae*. In a previous study involving six hospitals in England, temocillin was used in the treatment of urinary tract and bloodstream infections caused by ESBL/AmpC-producing *Enterobacteriaceae* with high clinical and microbiological cure rates (of 91% and 92% respectively) [5]. Temocillin was given 2 g twice daily irrespective of the ESBL/AmpC production by the infecting organism(s), reaffirming its potential application as a carbapenem-sparing agent [5].

There is a paucity of susceptibility data for temocillin in the Asian Pacific region. A Korean study revealed a temocillin susceptibility rate of 96.8% (335 out of 346) in *E. coli* from community-acquired urinary tract infection (UTI) [13], whereas in another study solely focusing on ESBL-producing *E. coli* from patients with community-acquired acute pyelonephritis [14], susceptibilities varied with 100% susceptibility (*N* = 11) in CTX-M-14 strains, while only 72.7% (*n* = 11) of CTX-M-15 strains were susceptible. In a recent study from Singapore, a high susceptibility rate of 95% for both *E. coli* and *K. pneumoniae* was reported when breakpoints for uncomplicated UTIs were applied; however, the rates were substantially lower when “systemic infection” breakpoints were used [15].

Currently, the resistance rates to agents such as ciprofloxacin, nitrofurantoin and piperacillin/tazobactam in Hong Kong are high, necessitating a switch to reserved agents such as colistin and fosfomycin for treating infections caused by MDRGNB. Temocillin may be a useful alternative for the treatment of infections caused by ESBL- and multi-drug-resistant *Enterobacteriaceae* in Hong Kong, particularly as a “carbapenem-sparing agent”. To our knowledge, this study is the first to report the use of the CLSI microbroth dilution method for the testing of temocillin and a good correlation of MIC values with temocillin between the BSAC and CLSI microbroth dilution methods was obtained.

## Electronic supplementary material

ESM 1 (DOCX 31 kb)
