# Supplementary material for: Activity of temocillin and 15 other agents, including fosfomycin and colistin, against Enterobacteriaceae in Hong Kong
Source: Eur J Clin Microbiol Infect Dis. 2017 Aug 25;36(12):2491–4. doi: 10.1007/s10096-017-3091-8 (PMC5688185; doi:10.1007/s10096-017-3091-8)
Supplement: Supplementary file 1 — (DOCX 31 kb) [file 10096_2017_3091_MOESM1_ESM.docx]

**Table 1 The minimum inhibitory concentrations, MIC_50_, and MIC_90_ of 16 antibiotics to 613 isolates of Enterobacteriaceae.**

| **Antimicrobial** | **MIC Range (µg/ml)** | **MIC Interpretative Criteria ( µg/ml)** | | | **MICs ( µg/ml)** | | | | |
| --- | --- | --- | --- | --- | --- | --- | --- | --- | --- |
|  |  | **S** | **I** | **R** | **%S (N)** | **%I (N)** | **%R (N)** | **MIC_50_** | **MIC_90_** |
| Temocillin all^*^ (N=613) | 0.5-64 | ≤8 | - | >8 | 93(570) | 0 | 7(43) | 4 | 8 |
| Temocillin^*^  (N=310 blood) | 0.5-64 | ≤8 | - | >8 | 91.9(285) | 0 | 8.1(25) | 4 | 8 |
| Temocillin^**^  (N=303 urine) | 0.5-64 | ≤32 | - | >32 | 100(303) | 0 | 0 | 4 | 8 |
| Ceftriaxone | 0.03-128 | ≤1 | 2 | ≥4 | 75.4(462) | 1.1(7) | 23.5(144) | 0.03 | 32 |
| Ceftazidime | 0.03-256 | ≤4 | 8 | ≥16 | 83.5(512) | 6.4(39) | 10.1(62) | 0.25 | 16 |
| Ertapenem | 0.015-128 | ≤0.5 | 1 | ≥2 | 99(607) | 0.5(3) | 0.5(3) | 0.015 | 0.06 |
| Meropenem | 0.015-128 | ≤1 | 2 | ≥4 | 99.7(611) | 0 | 0.3(2) | 0.015 | 0.06 |
| Gentamicin | 0.03-256 | ≤4 | 8 | ≥16 | 78.3(480) | 1.1(7) | 20.6(126) | 0.5 | 64 |
| Amikacin | 0.25-512 | ≤16 | 32 | ≥64 | 99.2(608) | 0 | 0.8(5) | 2 | 4 |
| Ciprofloxacin | 0.015-128 | ≤1 | 2 | ≥4 | 71.5(438) | 1(6) | 27.5(169) | 0.25 | 32 |
| Piperacillin/  tazobactam | 0.06/4-512/4 | ≤16/4 | 32/4- 64/4 | ≥128/4 | 86(527) | 12.4(76) | 1.6(10) | 4 | 32 |
| Amoxicillin/  clavulanate | 0.5/0.25-128/64 | ≤8/4 | 16/8 | ≥32/16 | 59.1(362) | 21.4(131) | 19.6(120) | 8/4 | 64/32 |
| Trimethoprim/  Sulfa+ | 0.06/1.19-128/2432 | ≤2/38 | - | ≥4/76 | 62.5(383) | 0 | 37.5(230) | 0.5/9.5 | >64/1216 |
| Nitrofurantoin^§^ | 0.5-128 | ≤32 | 64 | ≥128 | 76.4(430) | 13.5(76) | 10.1(57) | 16 | >64 |
| Cefepime | 0.03-128 | ≤2 | 4/8 | ≥16 | 81.1(497) | 9.3(57) | 9.6(59) | 0.125 | 8 |
| Fosfomycin^#^ | 0.12-1024 | ≤64 | 128 | ≥256 | 92.8(569) | 2.5(15) | 4.7(29) | 1 | 32 |
| Colistin^##§^ | 0.06-128 | ≤2 | 4 | ≥8 | 88.8(500) | 0.9(5) | 10.3(58) | 0.25 | 64 |
| Tigecycline^***^ | 0.015-16 | 1 | 2 | 2 | 548 (89.4) | 10.6(65) | 10.6(65) | 0.25 | 2 |
| ESBL status^++^ | Pos/total | 118/508 (23.2%) | | | |  | |  | |

^*^ BSAC method and systemic breakpoint,

^**^ BSAC method and urinary breakpoint,

+trimethoprim/sulfamethoxazole,

^#^performed according to CLSI with glucose-6-phosphate suppl.

^##^breakpoints according to CLSI for non-fermenters (*Pseudomonas aeruginosa*),

***interpreted with EUCAST breakpoints,

^++^ESBL testing inc. *E.coli, Klebsiella* spp, *Proteus* spp only

^§^ Total nos tested (563) to exclude intrinsically resistant *Proteus* spp.

**Table 2 The minimum inhibitory concentrations, MIC_50_, and MIC_90_ of 16 antibiotics to commonly isolated *Enterobacteriaceae*.**

| **Antimicrobial** | **MIC (µg/ml) in blood (153) and urine (196)** | | | | | | | | | | | | |
| --- | --- | --- | --- | --- | --- | --- | --- | --- | --- | --- | --- | --- | --- |
|  | **%S (N)** | | |  | **%I (N)** | | **%R (N)** | | **MIC_50_  (µg/ml)** | | **MIC_90_ (µg/ml)** | |  |
| ***E. coli*** (349) |  | | |  |  | |  | |  | |  | |  |
| Temocillin systemic^*^ | 88.2(135) | | |  | 0 | | 11.8(18) | | 4 | | 16 | |  |
| Temocillin urinary^**^ | 100(196) | | |  | 0 | | 0 | | 4 | | 8 | |  |
| Meropenem | 99.7(348) | | |  | 0 | | 0.3(1) | | 0.015 | | 0.015 | |  |
| Gentamicin | 69.1(241) | | |  | 0.9(3) | | 30(105) | | 1 | | 64 | |  |
| Ciprofloxacin | 63.9(223) | | |  | 0.9(3) | | 35.1(123) | | 0.25 | | 64 | |  |
| Piperacillin/tazobactam | 86.8(303) | | |  | 11.4(40) | | 1.7(6) | | 8 | | 32 | |  |
| Amoxicillin/clavulanate | 61.4(215) | | |  | 29.1(102) | | 9.2(32) | | 8/4 | | 16/8 | |  |
| Nitrofurantoin | 96.8(338) | | |  | 2.3(8) | | 0.9(3) | | 8 | | 16 | |  |
| Fosfomycin^#^ | 97.1(339) | | |  | 0.9(3) | | 2.0(7) | | 1 | | 2 | |  |
| Colistin^##^ | 97.4(341) | | |  | 1.1(4) | | 1.1(4) | | 0.25 | | 0.5 | |  |
| Tigecycline^**^ | 99.7(348) | | |  | 0.3(1) | | 0.3(1) | | 0.25 | | 0.5 | |  |
| ESBL pos | 99/349(28.4%) | | |  |  | |  | |  | |  | |  |
|  |  | | |  |  | |  | |  | |  | |  |
| ***Klebsiella* spp** (109) |  | | |  |  | |  | |  | |  | |  |
| Temocillin systemic^*^ | 98.3(57) | | |  | 0 | | 1.7(1) | | 2 | | 8 | |  |
| Temocillin urinary^**^ | 100(51) | | |  | 0 | | 0 | | 2 | | 16 | |  |
| Meropenem | 99.1(108) | | |  | 0 | | 0.9(1) | | 0.015 | | 0.06 | |  |
| Gentamicin | 90.8(99) | | |  | 1.8(2) | | 7.3(8) | | 0.25 | | 1 | |  |
| Ciprofloxacin | 78.9(86) | | |  | 0 | | 21.1(23) | | 0.015 | | 64 | |  |
| Piperacillin/tazobactam | 82.6(90) | | |  | 15.6(17) | | 1.8(2) | | 4 | | 32 | |  |
| Amoxicillin/clavulanate | 74.3(81) | | |  | 11(12) | | 14.7(16) | | 4/2 | | 64/32 | |  |
| Nitrofurantoin | 30.3(33) | | |  | 37.6(41) | | 32.1(35) | | 64 | | >64 | |  |
| Fosfomycin^#^ | 96.3(105) | | |  | 1.8(2) | | 1.8(2) | | 8 | | 32 | |  |
| Colistin^##^ | 99.1(108) | | |  | 0 | | 0.9(1) | | 0.25 | | 0.5 | |  |
| Tigecycline^**^ | 90.8(99) | | |  | 9.2(10) | | 9.2(10) | | 0.5 | | 1 | |  |
| ESBL pos | 17/106 | | |  |  | |  | |  | |  | |  |
| ***Enterobacter* spp** (35) | | |  | |  |  | |  | |  | |  |  |
| Temocillin systemic^*^ | | 84.6(22) | | | 0 | 15.4(4) | | 2 | | 16 | |  |  |
| Temocillin urinary^*^ | | 100(9) | | | 0 | 0 | | 4 | | 16 | |  |  |
| Meropenem | | 100(35) | | | 0 | 0 | | 0.06 | | 0.12 | |  |  |
| Gentamicin | | 97.1(34) | | | 0 | 2.9(1) | | 0.25 | | 0.5 | |  |  |
| Ciprofloxacin | | 100(35) | | | 0 | 0 | | 0.015 | | 0.25 | |  |  |
| Piperacillin/tazobactam | | 62.9(22) | | | 11 | 5.7(2) | | 4 | | 64 | |  |  |
| Amoxicillin/clavulanate | | | 5.7(2) | | 0 | 94.3(33) | | 64/32 | | >64/32 | | |  |
| Nitrofurantoin | | | 51.4(18) | | 12 | 14.3(5) | | 32 | | >64 | | |  |
| Cefepime | | | 94.3(33) | | 2 | 0 | | 0.12 | | 1 | | |  |
| Fosfomycin^#^ | | | 91.4(32) | | 1 | 5.7(2) | | 16 | | 64 | | |  |
| Colistin^##^ | | | 65.7(23) | | 0 | 34.3(12) | | 0.25 | | >64 | | |  |
| Tigecycline^**^ | | | 100(35) | | 0 | 0 | | 0.5 | | 0.5 | | |  |
| ESBL pos | | |  | |  |  | |  | |  | | |  |
| All ESBL positive (118) | | |  | |  |  | |  | |  | | |  |
| Temocillin (118)^*^ | | | 83.9(118) | | 0 | 16.1(19) | | 8 | | 16 | | |  |
| Temocillin (blood)^*^ | | | 83.3(60) | | 0 | 16.7(10) | | 8 | | 16 | | |  |
| Temocillin (urine)^**^ | | | 100(58) | | 0 | 0 | | 8 | | 16 | | |  |

^*^ BSAC method and systemic breakpoint,

^**^ BSAC method and urinary breakpoint.
